# Effects of Carbohydrate Mouth Rinsing on Salivary Lysozyme, Mood States and Running Performance Among Recreational Runners

**DOI:** 10.21315/mjms2020.27.1.9

**Published:** 2020-02-27

**Authors:** Ayu Suzailiana Muhamad, Nurul Fatin Raihan Mohd Puad, Garry Kuan

**Affiliations:** Exercise and Sports Science Programme, School of Health Sciences, Universiti Sains Malaysia, Kelantan, Malaysia

**Keywords:** mouth rinsing, antimicrobial proteins, Brunel mood scale, exercise, recreational athletes

## Abstract

**Introduction:**

Carbohydrate (CHO) mouth rinsing can enhance sports performance through a central action mediated by receptors in the mouth. This study examined the effect of a CHO mouth rinse on salivary lysozyme concentrations, mood states and running performances.

**Methods:**

Ten males recreational runners were randomised to three running trials with a 1 week recovery period between the trials. Each trial involved running at 75% maximum heart rate (HRmax) for 1 h, followed by a 15 min time trial. The participants used a CHO mouth rinse, placebo (PLA) solution or control (CON, no solution) every 15 min during the exercise. Heart rate (HR), rating of perceived exertion (RPE) and mood states were recorded pre-, during and post-exercise. Saliva samples were collected pre-, post- and 1 h post-exercise.

**Results:**

There was no significant interaction and time effect (*P* > 0.05) on the salivary lysozyme concentration and running performance, but it was significant (*P* < 0.05) for HR and RPE (increase in all trials). However, there was no significant difference (*P* > 0.05) in salivary lysozyme concentrations, running performances, HR values or RPE between the trials. Mood states were not significantly different (*P* > 0.05) between the trials, but one of the mood sub-scales showed a significant (*P* < 0.001) time effect (increase fatigue in all trials).

**Conclusion:**

CHO mouth rinsing did not affect physiological parameters, salivary lysozyme concentrations, mood states or running performance among recreational runners.

## Introduction

Carbohydrate (CHO) intake during exercise can delay the onset of fatigue and improve performance during prolonged exercise (1). However, ingesting too much CHO can have detrimental effects. For example, highly concentrated CHO solutions and drinks with high osmolality have been linked to the development of gastrointestinal discomfort (2). As CHO is not swallowed during mouth rinsing, this might be a good strategy to improve sports performance without inducing gastrointestinal discomfort during exercise. The first study in this area by Carter et al. (3) reported that rinsing with a CHO mouthwash may have beneficial effects during high-intensity exercise. Several later studies reported that CHO mouth rinsing improved both cycling (4) and running (5) performances. In addition, CHO mouth rinsing increased the total distance covered during a self-selected 30 min run (6) and a 60 min self-paced run (7) in comparison to a placebo (PLA) mouth rinse.

The mechanism by which CHO mouth rinsing increases sports performance remains unclear. However, according to previous research, the mechanism may involve a group of receptors in the oral cavity with connections to reward areas in the brain (8). Based on this idea, activation of these areas in the brain leads to a reduced perception of exertion during exercise (4) and possibly reduced feelings of discomfort (9). Some evidence suggests that the magnitude of performance improvements with CHO mouth rinsing may be dependent on several factors, including the duration of fasting (10) and time of mouth rinsing (11). Based on this premise, the physiological status of the body, such as a fasted or fed state (4), could alter the central response to detecting CHO in the mouth. There is also evidence that the duration of mouth rinsing is important, with rinsing for 10 sec better than 5 sec in terms of exercise performance, suggesting a dose response to the duration of mouth rinsing (11).

Several studies reported that athletes who performed intensive and prolonged exercise had an elevated risk of minor illnesses, particularly upper respiratory tract infections (12, 13). However, to date, no published studies have reported the effects of CHO mouth rinsing on mucosal immunity and mood states in response to exercise. As mucosal immunity in association with innate non-specific defence acts as the first line of defence against pathogens, allergens and antigen presentation (14), it would be interesting to investigate whether CHO mouth rinsing might enhance mucosal immunity among athletes. In addition, as previous studies reported that the perception of exertion and feelings of discomfort declined with CHO mouth rinsing (4, 9), it would be interesting to determine whether CHO mouth rinsing improved athletes’ mood states. Hence, the purpose of this study was to investigate the effects of CHO mouth rinsing on mucosal immunity, mood states and running performance among recreational runners.

## Methods

### Research Design and Randomisation

In this randomised, placebo-controlled, double-blinded and cross-over study, the participants were randomised to three running trials: CHO, PLA or control (CON). The recovery period between the trials was 1 week. The participants were randomised using computer generated randomised software (www.randomize.net). The CON trial did not involve mouth rinsing. In the CHO and PLA trials, the participants were given a plastic cup containing 25 mL of CHO or PLA, which they used as a mouth rinse every 15 min during the exercise. This study was conducted at the Sports Science Laboratory, Universiti Sains Malaysia (USM), Kelantan.

### Participants

The sample size was calculated by using PS Power and Sample Size Calculation version 3.0.43. Based on a previous study (11), the power of study was set at 80% with 95% confident interval, the standard deviation (SD) observed was 1.2 km of cycling performance and the difference in population mean was set at 1.16 km of cycling performance. The calculated sample size was 10 participants.

Students (*N* = 10) attending USM were recruited using the convenience sampling method via a poster placed on notice boards in the campus. The selection criteria were: male sex, recreational athlete, healthy, aged 18–26 years old and a non-smoker. The health status of the participants was assessed during the recruitment process using the Physical Activity Readiness Questionnaire (PAR-Q) form (available online). Only those who answered ‘No’ to all the questions in the first section of the questionnaire were recruited. Throughout the study period, the participants abstained from taking any supplements known to affect immune function.

### Research Procedures

The participants came to the laboratory at 8.30 a.m. after an overnight fast from 11.00 p.m. (plain water was permitted during this fasting period). Each participant’s body weight was measured and the first saliva sample was collected by 5 min un-stimulated dribbling into a sterile bijou tube. Each participant was asked to sit on a chair, lean their head forward and let the saliva passively dribble into the tube, without using any tongue or mouth movement.

Subsequently, the participants were asked to complete the Brunel mood scale (BRUMS) to provide an assessment of mood states (15). The BRUMS is a validated 24-item questionnaire. It contains six sub-scales, with each of the subscales containing four mood descriptors. The sub-scales are anger, confusion, depression, fatigue, tension and vigour. The BRUMS was validated and translated into the Malay language by Hashim et al. (16) in a study on 355 young Malaysian athletes, with alpha coefficients ranging from 0.72, 0.64, 0.73, 0.69, 0.65 and 0.58 for tension, depression, anger, vigour, fatigue and confusion, respectively.

After completing the BRUMS, the participants performed the running trial (CHO, PLA or CON). Each trial started with a warm-up by running on a motorised treadmill at 50% of maximum heart rate (HRmax) for 5 min, followed by running for another 60 min at 75% HRmax. The CON trial did not involve mouth rinsing. For CHO and PLA trials, at every 15 min, participants used CHO or PLA solution, respectively, as a mouth rinse for 10 sec before expectorating back into the cup. Immediately following the 60 min run, the participants were asked to run as fast as they could in 15 min (time trial) whereby they can adjust their running speed as desired. The running distance covered during this 15 min time trial was recorded.

The resting HR and rating of perceived exertion (RPE) were recorded before and after the warm-up, during the trial and after the time trial. Second and third saliva samples were collected upon finishing the time trial and 1 h post-exercise. Post-exercise, all the participants completed the BRUMS again.

### Preparation of CHO and Placebo Solutions

The CHO solution was prepared by adding 64 g of glucose into 1000 mL of water (4). The PLA solution was prepared by adding 17 g of commercially available non-caloric sweetener stevia into 1000 mL of water. Its colour and taste were similar to the CHO solution but contained no carbohydrate. Both solutions were prepared and labelled by a laboratory staff to ensure double blinding.

### Saliva Samples Analysis

Saliva samples were centrifuged at 12,000 rpm for 10 min by using a centrifuge. Its liquid part was transferred into a labelled tube and kept in the freezer at −20 °C until further analysis of salivary lysozyme was carried out by using a commercially available reagent kit via the ELISA method.

### Statistical Analysis

By using Statistical Package for Social Science version 23.0, the normality of all the data was examined through the Kolmogorov-Smirnov test. One-way analysis of variance (ANOVA) was used to analyse differences of participants’ age, body weight, height and BMI between trials. Two-way ANOVA with repeated measures was performed to determine the significance of the differences of HR, RPE, lysozyme concentration and BRUMS scores between trials and within each trial. The difference was considered statistically significant at *P* < 0.05. All values were presented as mean (standard deviations).

## Results

### Physiological Characteristics of the Participants

Mean age, weight, height and body mass index (BMI) of all the 10 participants are shown in Table 1. The values were analysed using descriptive statistics. One-way ANOVA analysis revealed that mean age, body weight, height and BMI were not significantly different (*P* > 0.05) between groups.

### Heart Rate (HR)

There was a significant interaction effect (*F*_(12,46)_ = 0.578, *P* = 0.018) and main effect of time (*F*_(6,22)_ = 289, *P* **<** 0.001) on HR values in the trials, with HR values significantly increasing in all trials from baseline to the end of the exercise. However, there was no significant difference in HR values among the trials (*F*_(2,27)_ = 0.475, *P* = 0.627). The average HR values at baseline and at the end of each trial are shown in Table 1.

### Rate of Perceived Exertion

There was a significant time × trial interaction effect (*F*_(12,46)_ = 0.514, *P* = 0.048) and main effect of time (*F*_(6,22)_ = 52, *P* < 0.001) on RPE in the trials, with RPE increasing significantly in all trials from baseline to the end of the exercise. However, there was no significant difference in RPE among the trials (*F*_(2,27)_ = 0.257, *P* = 0.775). Table 1 shows the average RPE values at baseline and at the end of each trial.

### Running Performance

**Figure 1:**
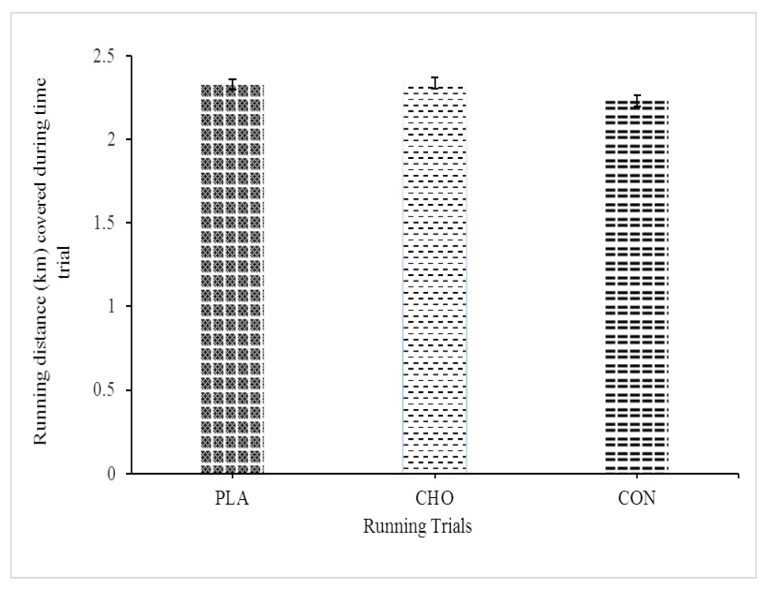
Running distance covered (km) during the 15 min time trial

There was no significant difference (*F*_(2,18)_ = 2.591, *P* = 0.103) in the running distance covered during the time trial in the three trials (Figure 1). The mean running distance covered in the CHO, PLA and CON (no mouth rinse) trials was 2.34, 2.33 and 2.23 km, respectively.

### Salivary Lysozyme Concentration

**Figure 2:**
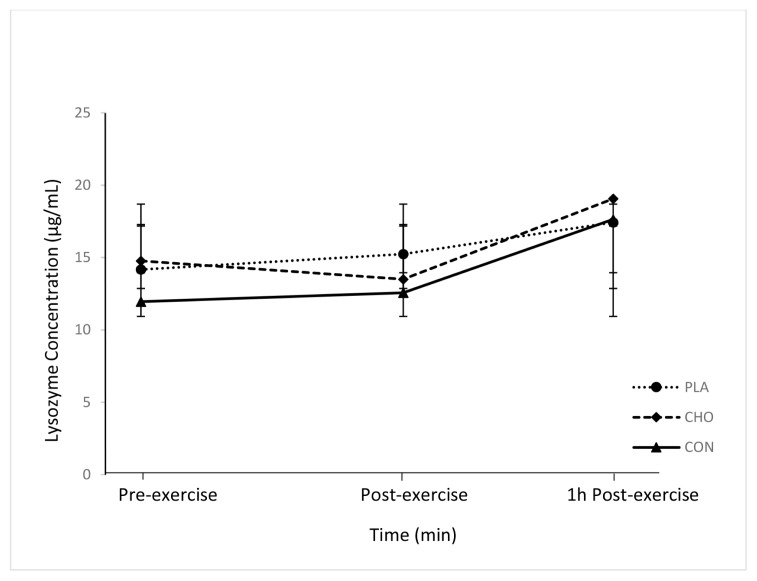
Lysozyme concentration (μg/mL) in all trials

There was no significant time × trial interaction effect (*F*_(4,54)_ = 0.101; *P* = 0.101) on salivary lysozyme concentration (Figure 2). There was also no significant main effect of time (*F*_(2,54)_ = 1.813, *P* = 0.173) on salivary lysozyme concentration during the trials and no significant difference (*F*_(2,27)_ = 0.141, *P* = 0.869) on lysozyme concentration among the trials.

### BRUMS

There was a significant time effect for fatigue (*F*_(1,27)_ = 18.43, *P* < 0.001) (Table 2) but not on other sub-scales. However, there was no significant interaction between time and trial on all sub-scales. In addition, there was also no significant difference on all sub-scales between trials.

## Discussion

### Heart Rate

In agreement with our findings, several other studies reported that HR increased progressively during exercise (4, 17). The increase in HR during exercise is due to the increased demand for oxygen supply to working muscles. Chronotropic and inotropic effects on the heart are stimulated by the noradrenergic sympathetic nervous system. In the present study, HR values were unaffected by mouth rinsing or the type of mouth rinsing solution used during prolonged exercise. Other studies reported similar observations (3, 17–19). The results suggest that a CHO mouth rinse during exercise does not affect HR and that the effect depends on the type, intensity and volume of the exercise (20).

### Rate of Perceived Exertion

Perceived exertion is defined as physical sensations experienced by an individual during physical activity (21). In the present study, RPE rose from 6 to 17 in the Borg’s scale in all the trials, showing that physical exertion perceived by the participants steadily increased over time, particularly at the end of the time trial in all the trials. Although perceived exertion is a subjective measurement, a person’s exertion rating may provide a fairly good estimate of the actual heart rate values during physical activity (21). The present study did not detect any statistically significant difference in RPE between the trials. Similarly, previous studies that reported a performance benefit from CHO mouth rinsing reported no difference in subjects’ perceived exertion (3, 4, 10, 22–24). In contrast, some studies (9, 19) reported lower RPE in a CHO mouth rinsing trial as compared with that in a PLA trial. The discordant findings might be attributed to different types of exercises and different exercise durations and intensities in the various studies.

### Running Performance

In this study, running performance was determined by the running distance covered by the participants during each time trial. The results of the statistical analysis revealed no significant difference in the time trial running distance covered in the three trials (Figure 1). This finding was similar to that of a previous study on the effect of a CHO mouth rinse or PLA on running performance (24). The authors detected no significant difference in the overall distance covered in a 45 min time trial in the CHO trial (6% CHO) and PLA trial. In contrast, another study reported that recreational runners who used a CHO mouth rinse covered a greater running distance than those who used a PLA mouth rinse (17). Similarly, researchers found positive effects of CHO mouth rinsing on cycling performance (3, 22), reporting that cyclists who used a CHO mouth rinse completed a time trial significantly faster than those who used a PLA mouth rinse. All the previous studies (3, 4, 22, 23, 25) that reported positive findings of a CHO mouth rinse on exercise performance involved higher intensity (≥ 75% of VO_2_max) exercise than that performed in the present study, in which the participants performed lower exercise intensity. It is possible that the exercise protocol in the present study did not induce sufficient exertion to gain possible benefits from CHO mouth rinsing.

### Salivary Lysozyme Concentration

In the present study, CHO mouth rinsing had no effect on salivary lysozyme concentrations (Figure 2). To date, there are limited reports on the effects of CHO mouth rinsing on salivary lysozyme concentrations. However, the salivary lysozyme concentration increased immediately after food consumption during the first hour of recovery after prolonged exercise in the CHO trial relative to that in the PLA trial (26). The increase in the lysozyme concentration during CHO feeding is associated with the secretion of saliva flow rate. The findings illustrate the importance of the saliva flow rate in the regulation of oral mucosal immunity (27–29).

In addition, this study found that the salivary lysozyme concentration was not significantly affected by the prolonged exercise in all the trials. A previous study on elite rowers (30) also observed no significant change in salivary lysozyme concentrations in response to exercise. This finding was inconsistent with that of some previous studies (31–34), which found an increase in salivary lysozyme secretion following exercise. In these studies, lysozyme responses increased significantly with moderate exercise intensity and increased further with high-intensity exercise (35). These findings suggest that the lysozyme secretion rate depends on the exercise intensity, where exercise intensity is directly proportional to the lysozyme secretion rate.

In general, exercise-induced alterations in mucosal secretions were largely dependent on the exercise intensity or duration. Higher intensity (> 70% VO_2_max) or longer duration (> 40 min) exercise appeared to elicit greater concentrations and secretion rates of anti-microbial polypeptides (34, 36, 37). For example, Allgrove et al. (34) reported significant increases in salivary lysozyme concentrations after exercise at 75% VO_2_max on a cycle ergometer. Similarly, Usui et al. (37) noted temporary increases in salivary lysozyme concentrations in 10 young male volunteers after prolonged strenuous exercise at 75% VO_2_max for 60 min. Davison et al. (36) reported significant increases in salivary lysozyme concentrations after 2.5 h of cycling exercise at 60% of VO_2_ max. In contrast, low-intensity or short-duration exercise did not appear to significantly change antimicrobial polypeptide responses compared to resting values (34). In a cycle ergometer exercise at 50% VO_2_max, non-significant changes in salivary lysozyme concentration were reported after exercise for approximately 22 min (34). Furthermore, West et al. (30) reported that exercise had no effect on salivary lysozyme concentrations.

According to previous research, increased secretion of lysozymes from salivary glands during exercise is a function of the accumulation of proteins over time (38). Some authors proposed that increased sympathetic nervous system activity may explain the acute increase in salivary lysozymes after high-intensity exercise (30). Others suggested that an increase in the concentration of salivary lysozymes after exercise might confer improved immunity to infection (29, 30). Chronic stress was shown to be associated with reduced secretion of salivary lysozymes (39). Hence, it could be suggested that prolonged intense exercise may have a negative effect on the concentration of lysozymes in the upper respiratory tract. Although prolonged intense exercise causes transient perturbations in mucosal immunity, the relative load or intensity of exercise required to produce such perturbations varies among individuals, depending on physical capacity, training history and fitness levels. These factors need to be considered when determining the intensity and duration to be applied in experimental settings (30). Thus, this inconsistency might confound the results of the study.

### BRUMS

To date, there is limited research on perceptual responses and mood during exercise. In the present study, we investigated mood responses following CHO, PLA and CON trials. CHO mouth rinsing did not affect mood states as assessed by the BRUMS. However, the exercise itself significantly increased feelings of fatigue but not on the other sub-scales (Table 2). Mood states fluctuate as a situation changes, and there is no ideal mood for superior performance (40). The researcher has indicated that there was a two-way relationship between mood and emotion. In the study, mood shaped athletes’ emotions, and subjective experience resulted in a particular mood state. Lane et al. (41) showed that depressed mood acted as a moderator of other manifestations of mood in athletic performance. In the presence of depression, increased levels of negative mood states, such as anger, tension, confusion and fatigue, had adverse effects on performance. Ali et al. (18) reported that mouth rinsing or ingestion did not influence the profile of mood scale (POMS) score. However, they observed an interaction effect for the ‘vigour’ sub-scale, with higher ratings over time in CHO mouth rinsing compared to a PLA mouth rinse and CHO ingestion trials. Rollo et al. (5) reported that mouth rinsing with a CHO solution significantly increased Feeling Scale ratings, with the CHO group having higher scores for pleasurable feelings immediately prior to a 30-min run than a PLA mouth rinse solution group. In a magnetic resonance imaging study, Chambers et al. (4) reported that CHO mouth rinsing enhanced motivation and activity of motor control centres. They suggested that CHO mouth rinsing could enhance mood by stimulating pleasure and reward centres in the brain. The activation of these brain regions may influence emotions and behaviour and consequently exercise performance (42). Despite positive findings of the effect of CHO mouth rinsing on mood in previous studies, CHO mouth rinsing did not significantly enhance mood states during prolonged exercise in the present study. We speculate that reduced stress induced by the exercise protocol employed in this study and the fitness level of the participants may have hindered the potential benefits of CHO mouth rinsing.

## Conclusion

It can be summarised that CHO mouth rinsing has no significant effects on the running distance covered during the time trial, HR, RPE, lysozyme concentration and mood states. However, it was noted that prolonged exercise significantly increased HR, RPE and fatigue subscale of mood. As a conclusion, the present study found that CHO mouth rinsing has no beneficial effects on physiological parameters, salivary lysozyme responses, running performance and mood states during prolonged exercise among recreational runners.

## Figures and Tables

**Table 1:** Physical and physiological characteristics of the participants

Parameters	Mean (SD)			*P*-value

	CHO	PLA	CON	
Age (years old)	21.2 (4.4)	22.2 (2.8)	22.0 (3.7)	0.442
Body weight (kg)	60.7 (11.8)	62.8 (8.5)	61.7 (5.6)	0.094
Height (cm)	165.8 (4.6)	166.8 (8.6)	164.8 (8.1)	0.167
Body mass index (kg/m^2^ )	22.1 (1.5)	22.6 (2.6)	22.7 (2.4)	0.318
HR-baseline (beats/min)	75.3 (3.4)	78.5 (4.4)	76.1 (4.6)	Time effect:*P* = 0.001
HR-post-trial (beats/min)	162.7 (1.5)	165.3 (2.4)	163.4 (3.7)	Group effect:*P* = 0.627
RPE-baseline (scale 6–20)	6.0 (0.0)	6.0 (0.0)	6.0 (0.0)	Time effect:*P* = 0.001
RPE-post-trial (scale 6–20)	15.2 (1.2)	15.8 (2.7)	14.5 (5.1)	Group effect:*P* = 0.775

**Table 2:** BRUMS scores in all trials

Variables	Groups	Pre-trial Mean (SD)	Post-trial Mean (SD)	Time Effect	Time^*^ Group	Group Effect

				*F*-stat (df) *P*-value	*F*-stat (df) *P*-value	*F*-stat (df) *P*-value
Vigour	PLA	60.6 (11.3)	53.4 (7.6)	1.51 (1,27)	0.91 (2,27)	0.01 (1,27)
	CHO	57.2 (13.1)	57.2 (7.1)	*P* = 0.293	*P* = 0.416	*P* = 0.996
	CON	57.6 (11.3)	57.0 (10.2)			
Anger	PLA	44.8 (1.6)	45.8 (4.4)	1.17 (1,27)	0.28 (2,27)	0.81 (1,27)
	CHO	44.4 (1.2)	44.7 (2.2)	*P* = 0.287	*P* = 0.757	*P* = 0.455
	CON	45.1 (2.4)	47.4 (8.5)			
Confusion	PLA	47.2 (4.2)	48.6 (10.9)	1.30 (1,27)	0.07 (2,27)	0.26 (1,27)
	CHO	44.8 (4.3)	48.0 (9.1)	*P* = 0.264	*P* = 0.936	*P* = 0.775
	CON	44.7 (3.3)	47.7 (13.0)			
Fatigue	PLA	48.4 (7.8)	56.9 (9.9)	18.43 (1,27)	0.25 (2,27)	0.03 (1,27)
	CHO	48.8 (6.2)	55.7 (9.2)	*P* < 0.001 ***	*P* = 0.784	*P* = 0.974
	CON	50.2 (9.6)	55.9 (10.6)			
Tension	PLA	54.8 (14)	51.4 (13.8)	0.03 (1,27)	0.24 (2,27)	1.50 (1,27)
	CHO	49.1 (9.5)	49.4 (10.6)	*P* = 0.861	*P* = 0.789	*P* = 0.243
	CON	46.8 (6.3)	48.3 (9.9)			
Depression	PLA	44.5 (2.5)	51.3 (14.8)	4.20 (1,27)	0.56 (2,27)	0.42 (1,27)
	CHO	44.5 (2.5)	47.5 (6.9)	*P* = 0.050	*P* = 0.581	*P* = 0.659
	CON	44.8 (8.9)	50.1 (11.9)			

Notes:

*** (*P* <0.001)-Significantly different betweenpre- and post-trial
